# More Instructions Make Fewer Subtractions

**DOI:** 10.3389/fpsyg.2021.720616

**Published:** 2021-09-28

**Authors:** Martin H. Fischer, Bodo Winter, Arianna Felisatti, Andriy Myachykov, Melinda A. Mende, Samuel Shaki

**Affiliations:** ^1^Cognitive Sciences Division, University of Potsdam, Potsdam, Germany; ^2^Department of English Language and Linguistics, University of Birmingham, Birmingham, United Kingdom; ^3^Department of Psychology, Northumbria University Newcastle, Newcastle upon Tyne, United Kingdom; ^4^Department of Behavioral Sciences, Ariel University, Ariel, Israel

**Keywords:** problem solving, addition, subtraction, cognitive bias, SNARC

## Introduction

Research on problem solving offers insights into how humans process task-related information and which strategies they use (Newell and Simon, 1972; Öllinger et al., 2014). Problem solving can be defined as the search for possible changes in one's mind (Kahneman, 2003). In a recent study, Adams et al. (2021) assessed whether the predominant problem solving strategy when making changes involves adding or subtracting elements. In order to do this, they used several examples of simple problems, such as editing text or making visual patterns symmetrical, either in naturalistic settings or on-line. The essence of the authors' findings is a strong preference to add rather than subtract elements across a diverse range of problems, including the stabilizing of artifacts, creating symmetrical patterns, or editing texts. More specifically, they succeeded in demonstrating that “participants were less likely to identify advantageous subtractive changes when the task did not (vs. did) cue them to consider subtraction, when they had only one opportunity (vs. several) to recognize the shortcomings of an additive search strategy or when they were under a higher (vs. lower) cognitive load” (Adams et al., 2021, p. 258).

Addition and subtraction are generally defined as de-contextualized mathematical operations using abstract symbols (Russell, 1903/1938). Nevertheless, understanding of both symbols and operations is informed by everyday activities, such as making or breaking objects (Lakoff and Núñez, 2000; Fischer and Shaki, 2018). The universal attribution of “addition bias” or “subtraction neglect” to problem solving activities is perhaps a convenient shorthand but it overlooks influential framing effects beyond those already acknowledged in the report and the accompanying commentary (Meyvis and Yoon, 2021).

Most importantly, while Adams et al.'s study addresses an important issue, their very method of verbally instructing participants, together with lack of control over several known biases, might render their findings less than conclusive. Below, we discuss our concerns that emerged from the identified biases, namely those regarding the instructions and the experimental materials. Moreover, we refer to research from mathematical cognition that provides new insights into Adams et al.'s findings.

## Bias From Language-Based Instructions

The first bias emerges from merely instructing participants to do something. This alone already implies addition: With the exception of specific subtraction terms like “remove pieces,” any socially embedded request to “make” or “do” (apart from specialized constructions like “make less of.”) demands creating and thus adding. Language statistics reflect this language-immanent hortatory bias, as the authors themselves demonstrate by regularly instructing their participants to “add or subtract” but never to “subtract or add”. This ordering preference makes adding more salient than subtracting. Moreover, in the control conditions of their first three experiments, the authors explicitly mentioned addition (“each piece that you add costs ten cents”) but not subtraction. In contrast, there was never an instruction in the control conditions that explicitly mentioned subtraction but not addition. This creates the context whereby mentioning “addition” first creates bias toward adding, because the first mentioned word is more salient (e.g., Zadeh, 1975).

Adams and colleagues' instructions actually reflect a general pattern of language use—there are 95 occurrences of “add and subtract” but zero occurrences of “subtract and add” in a representative English language corpus^1^. A distributional semantic analysis of the verbs used in their instructions reveals an additional dimension of bias: A Latent Semantic Analysis (Deerwester et al., 1990) reveals that the supposedly neutral terms “improve,” “arrange” and “change” are all more strongly associated with “addition” (as indicated by similarity scores of 0.22, 0.33 and 0.22, respectively) than with subtraction (0.03, 0.14, and 016, respectively.^2^

Overall, we believe that linguistic instruction, different from other cognitive, cultural and socioecological factors already considered by the authors and commentators, the language-based nature of instruction constitutes a separate, more profound and indeed overlooked source of bias. While the authors documented differences between conditions that identified a subtraction neglect, our linguistic argument draws attention to the ignored baseline value of addition bias in the language.

The authors might argue that, when comparing a “no cue” to a “with cue” condition, the added instructional cue “add and subtract” should lead to more additions under our perspective but instead resulted in more subtractions (in their Experiments 2–4). However, our “biased language argument” implies that the “no cue” condition is actually not neutral and already contains the “addition” bias. Thus, adding the instructional cue adds only a single new element, namely “subtraction.” Consistent with the semantic priming literature (e.g., Mandera et al., 2017), this change draws attention and leads to the corresponding behavioral compliance observed.

## Bias From Stimulus Design

The overall study by Adams and colleagues deserves strong acknowledgment because a large number of demonstrations from a variety of domains consistently points in the same direction, suggesting that we neglect subtractions in favor of additions. Nevertheless, it is also true that the power of this conclusion depends on the strength of the individual component arguments. We therefore point to several limitations of the current evidence that are associated with the experimental materials used in the study. For example, participants saw a Lego^TM^ Tower that had on its top a single lateralized stone that barely supported a flat roof. Their task was to modify this construction to enable the secure placement of a brick on top of the roof. The authors placed a Lego^TM^ figure in front of this tower construction (see Figure 2 of Adams et al., 2021). This figure will have drawn participants' attention to the front of the tower, where only adding a block was possible, thereby either signaling that the extra height of the platform was not necessary, or perhaps reducing the probability of subtracting the superfluous block in the back of the tower.

In another problem, participants were provided with a set of four grids of 10 × 10 squares that were either white or green. Their task was to make the green area symmetrical by toggling green squares to white or white squares to green per mouse click. Importantly, all four toggle grids initially contained more white than green fields (first grid; 73 white vs. 27 green squares; second: 54 vs. 46; third: 68 vs. 32; fourth 56 vs. 44). Thus, the authors implemented 63% white and 37% green squares; this design feature biased performance against subtraction because there were already more white squares (see Figure 1 of Adams et al., 2021). Importantly, the entire argument hinges on what is perceived to be the figure and what is perceived to be the ground because, in principle, a toggle operation is neither adding nor subtracting anything unless a target or a figure object has been defined. Aside from the mere frequency advantage that makes the green pattern the figure, it remains unclear whether each entire grid was placed against a white background (just as it appears in the printed article). Although this criticism does not account for the effect of cognitive load (which magnified subtraction neglect) an interesting prediction is that, on a green page, the opposite preference might emerge for the same patterns.

## Insights From Mathematical Cognition Research

Third, Adams et al. (2021) reported participants' sensitivity to instruction manipulations. This insight converges with findings obtained from mathematical cognition research. To begin with, arithmetic operations are spatially associated: the widely spread population stereotypes have addition associated with right/up, while subtraction is associated with left/down (Winter et al., 2015). Adams et al.'s materials were not controlled for these pre-experimentally existing associations. One example is the golf hole depiction used to elicit modifications (see Figure 3 in Adams et al., 2021). The picture implies progression toward the upper right corner, thus inviting addition as the preferred associated response.

Furthermore, a key requirement for fair cognitive comparison of arithmetic operations is results-matching (Shaki et al., 2018). Empirical aesthetics has found that people have a preference for more complex as opposed to simple arrangements (Kaplan et al., 1972; Jacobsen and Höfel, 2002), making it problematic that all “addition” outcomes in the Adams et al. (2021) report were visually more complex than “subtraction” outcomes. Evaluating arithmetic preferences in a purely mathematical task that requires participants to create arithmetic addition or subtraction facts from visually presented components (operands, operators, and results) could be used to avoid this complexity confound. This is illustrated in the study by Werner et al. (2019), which revealed no addition bias but confirmed the well-known sensitivity of operation choices to spatial cues.

## Future Studies

In order to clarify the issue of whether there is indeed a “subtraction neglect” or an “addition bias” in human problem solving, we suggest improvements to the experimental design of future studies in the two following ways.

Firstly, the experimental tasks can be improved by counterbalancing instructions and stimuli design. So, future studies should use both addition-biasing and subtraction-biasing instructions (and not just bias toward addition). One needs to be careful to create instructions that are not implicitly biasing toward addition, as could be assessed via Latent Semantic Analysis (as demonstrated above). Furthermore, presented stimuli should be extended by pictures that lead to “promoting” subtraction as well (for instance a golf hole that is directed from the top right to the bottom left). Finally, stimuli's visual complexity should be controlled by using novel versions of the two-dimensional toggle grids.

Secondly, we recommend investigating “addition bias” within a simpler domain first. Note that in the reported studies, processing domains were mixed; i.e., they required processing of complex shapes (2-dimensional grids), schema-like objects (golf hole), and three-dimensional objects (Lego^TM^ tower) as well as mental addition- and subtraction-like processes. Instead, we suggest leaving out complex stimuli and using a simple, one-dimensional task. A concrete experiment could consist of a line-modification task where participants are given counterbalanced instructions either to make asymmetrically divided lines symmetrical or symmetrically divided lines asymmetrical—either by adding or subtracting (subtracting or adding) elements to either side of the line. Such tasks would neither be confounded by cross-domain associations nor by the complexity of experimental materials. In contrast to the experiments reported in Adams et al. (2021), different spatial dimensions should be assessed separately (horizontal, vertical, sagittal). Also, spatial alignment of the line itself (asymmetrical to the left/to the right) should be controlled to eliminate task-induced spatial biases.

To summarize our commentary, we believe that a baseline distorted by using biased instructions, spatially unbalanced materials and procedures, as well as comparing across differentially complex outcomes considerably weakens the conclusions suggested by Adams et al. (2021). On the other hand, the bulk of converging evidence from conceptual replications and the direction of effects in favor of addition over subtraction is intriguing and we are grateful for their thought-provoking study that promotes deeper insights into the nature of our problem solving minds.

## Author Contributions

All authors made substantial conceptual contributions to this text and approved its submission.

## Funding

MF, AF, and SS acknowledge grant DFG-FI-1915/8-1 Competing heuristics and biases in mental arithmetic. AM acknowledges support from the Basic Research Program of the National Research University Higher School of Economics.

## Conflict of Interest

The authors declare that the research was conducted in the absence of any commercial or financial relationships that could be construed as a potential conflict of interest.

## Publisher's Note

All claims expressed in this article are solely those of the authors and do not necessarily represent those of their affiliated organizations, or those of the publisher, the editors and the reviewers. Any product that may be evaluated in this article, or claim that may be made by its manufacturer, is not guaranteed or endorsed by the publisher.

## Footnotes

^1^ https://www.english-corpora.org/coca/

^2^ http://lsa.colorado.edu/
